# Use of alpha-1 adrenoceptor antagonists in patients who underwent low-dose-rate brachytherapy for prostate cancer - a randomized controlled trial of silodosin versus naftopidil -

**DOI:** 10.1186/s13014-014-0302-7

**Published:** 2014-12-29

**Authors:** Nobumichi Tanaka, Kazumasa Torimoto, Isao Asakawa, Makito Miyake, Satoshi Anai, Akihide Hirayama, Masatoshi Hasegawa, Noboru Konishi, Kiyohide Fujimoto

**Affiliations:** Departments of Urology, Nara Medical University, 840 Shijo-cho, Kashihara, Nara 634-8522 Japan; Radiation Oncology, Nara Medical University, Kashihara, Nara 634-8522 Japan; Departments of Urology and Pathology, Nara Medical University, Kashihara, Nara 634-8522 Japan; Department of Urology, Nara Hospital Kinki University Faculty of Medicine, Ikoma, Nara 630-0293 Japan

**Keywords:** Prostate cancer, LDR-brachytherapy, Alpha-1 adrenoceptor antagonist, Urinary morbidity, Randomized controlled study

## Abstract

**Background:**

To evaluate the effect of two different alpha-1 adrenoceptor antagonists on lower urinary tract symptoms in patients who underwent LDR-brachytherapy.

**Methods:**

A total of 141 patients who had been clinically diagnosed with localized prostate cancer and underwent LDR-brachytherapy were enrolled. Patients were randomized and allocated to two groups (silodosin 8 mg vs. naftopidil 75 mg). The primary endpoint was a change in the international prostate symptom score (IPSS) at 3 months after seed implantation. Secondary endpoints included the recovery rate of IPSS at 12 months after seed implantation, the change in IPSS and overactive bladder symptom score, uroflowmetric parameters, and frequency volume chart (FVC). To determine independent variables that can predict IPSS recovery, logistic regression analysis was carried out.

**Results:**

The mean change in the IPSS at 3 months after seed implantation in both groups was ⊿10.6 (naftopidil) and ⊿10.4 (silodosin), respectively. There was not a significant difference between the two groups (p=0.728). An increase in urinary frequency and a decrease in total urinated volume and mean voided volume were observed in FVC for 12 months after seed implantation. Multivariate analysis revealed that the urethral dose (UD30) was an independent predictive parameter of IPSS recovery. Patients with UD30 < 200Gy showed a higher recovery rate of IPSS at 12 months after seed implantation.

**Conclusion:**

There was no significant difference of serial change in IPSS between silodosin and naftopidil during the first year after seed implantation. A lower dose on the urethra was an independent predictor of IPSS recovery at 12 months after seed implantation.

## Background

Low-dose-rate brachytherapy (LDR-brachytherapy) is one of the curative treatment modalities alongside radical prostatectomy or intensity modulated radiation therapy (IMRT) [1-5]. The most common adverse events of LDR-brachytherapy are urinary frequency and urgency [6-8]. More than 50% patients show urinary frequency and urgency for 6 months after seed implantation. Although an alpha-1 adrenoceptor antagonist is used to prevent and relieve these symptoms, the efficacy on these adverse events has not been assessed sufficiently. Three subtype alpha-1 adrenoceptors are alpha-_1A_, alpha-_1B_ and alpha-_1D_. Of these different subtypes, alpha-_1A_ and alpha-_1D_ receptors are predominantly found in the bladder and prostatic urethra [9]. Alpha-1 adrenoceptor antagonist is commonly used to improve the urinary condition of benign prostatic hypertrophy (BPH). Among several alpha-1 adrenoceptor antagonists, silodosin has a predominant affinity for the alpha-_1A_ subtype receptor, while naftopidil has affinity for the alpha-_1D_ subtype receptor. Patients who undergo LDR-brachytherapy commonly receive an alpha −1 blocker to prevent and treat their urinary adverse events.

In this study we evaluate the efficacy of two alpha-1 adrenoceptor antagonists for subtypes _1A_ and _1D_ in patients who underwent LDR-brachytherapy and present the urinary adverse events.

## Methods

Of 170 patients who were clinically diagnosed with localized prostate cancer (cT1c-2cN0M0) and underwent LDR-brachytherapy between July 2007 and April 2010, the 141 patients who provided written informed consent were enrolled in this prospective randomized study. Patients were randomized to be allocated to two antagonist treatment groups (silodosin: 8 mg per day vs. naftopidil: 75 mg per day) by irradiation modality (seed alone vs. boost), neoadjuvant androgen deprivation therapy (ADT), and pretreatment international prostate symptom score (IPSS) as an adjustment factor. Medication continued for at least 3 months after seed implantation, or until the International Prostate Symptom Score (IPSS) returned to the pretreatment score or less.

Patients in the monotherapy group were treated by seed implantation alone at a prescribed dose of 160 Gy, while patients who received external beam radiation therapy (EBRT) in combination were treated at a prescribed dose of 110 Gy. The target portion of EBRT was determined one month after seed implantation, and the patients received 45 Gy (in 25 fractions of 1.8 Gy per fraction) using 10 MV photon energy. The clinical target volume included both the entire prostate and the proximal third of the seminal vesicles.

The primary endpoint was IPSS at 3 months after seed implantation. The secondary endpoints were recovery rate of IPSS at 12 months after seed implantation, IPSS and overactive bladder symptom score (OABSS), maximum flow rate (Qmax) and voided volume and post voided residual (PVR) on uroflowmetry, and 24-hour urinary frequency and total and mean voided volume on a frequency volume chart (FVC) [10]. Each score was evaluated before seed implantation and at 1, 3, 6, and 12 months later. Recovery was defined as a return of the IPSS to the pretreatment score or less.

The IPSS recovery rate was calculated using a Kaplan-Meier curve. The difference was evaluated by the log-rank test. The serial change of each parameter was tested between the pretreatment score and each measured period by Wilcoxon signed rank test. The difference in inter-group comparison was tested by the Mann–Whitney U test. Sample size calculations determined that 62 patients in each group would be needed to detect at least a 25% difference of efficacy between the two study groups with α equal to 0.05 and power equal to 80%. The baseline characteristics between the two groups were tested by the chi-square test for categorical variables and the Mann–Whitney U test for continuous variables.

To elucidate the predictive parameters for return of IPSS to the baseline level at 12 months after seed implantation, we conducted both univariate and multivariate logistic regression analyses (stepwise selection method) using clinical parameters (age, neoadjuvant ADT, combination of EBRT, adjuvant ADT, prostate volume at post-dosimetry, IPSS at baseline, alpha-1 adrenoceptor antagonist) and postoperative dosimetric parameters. The dosimetric parameters analyzed in this study were minimal percentage of the dose and minimal dose (Gy) received by 90% of the prostate gland (%D90/D90), percentage of the prostate volume receiving 100% and 150% of the prescribed minimal peripheral dose (V100/150), minimal percentage of the dose and minimal dose (Gy) received by 30% of the urethra (%UD30/UD30), minimal percentage of the dose and minimal dose (Gy) received by 90% of the urethra (%UD90/UD90), rectal volume (mL) receiving 100% of the prescribed dose (R100) and biologically effective dose (BED). BED was calculated to evaluate an independent factor to predict IPSS return, and an α/β ratio of 2 was used. The BED values of both seed implantation and EBRT were summed for patients treated with EBRT. Post-implant CT scanning and post-implant dosimetric studies were performed by one radiation oncologist (A.I.) at 1 month after seed implantation. The parameters that showed univariate significance (p-value of less than 0.10) were input into multivariate models.

All statistical analyses were performed using PASW Statistics 17.0 (SPSS Inc., Chicago, IL, USA). All *p* values of less than 0.05 were considered statistically significant. The institutional review board approved this prospective study, and informed consent was obtained from all patients after explaining the aim and methods of this study.

## Results

The patient characteristics are shown in Table 1. Each variable of patient characteristics did not show significant differences between the naftopidil and silodosin groups. Among all 141 patients, there were 4 (2.8%) who suffered acute urinary retention requiring an indwelling urethral catheter (naftopidil group: 2 patients, silodosin group: 2 patients). There was not a significant difference in the incidence of acute urinary retention between the two groups, and all patients recovered from acute urinary retention.

**Table 1:** **Patients characteristics**

**Variables**	**Naftopidil (n=70)**	**Silodosin (n=71)**	**p-value**
Age median (range)	72 (56–83)	70 (57–83)	0.216*
PSA (ng/mL) median range	6.5 (3.7-31.8)	7.2 (3.5-17.7)	0.421*
Duration of NeoADT (month) median (range)	4 (2–43)	4 (1–34)	0.640*
Prostate volume (mL) at impant median (range)	26 (8–42)	27 (10–48)	0.187*
IPSS at baseline median (range)	6 (0–17)	7 (0–23)	0.132*
OABSS at baseline median (range)	3 (0–8)	3 (0–10)	0.343*
No of needle/seeds median (range)	22 (15–30)/60 (40–90)	22 (16–30)/65 (35–90)	0.304/0.128*
Total activity (MBq) median (range)	875 (449–1314)	876 (438–1314)	0.221*
Stage T1c/T2a/T2b/T2c/T3a	38/25/6/1/0	37/28/4/1/1	0.813^†^
Gleason score −6/7/8-10	44/24/2	45/24/2	0.648^†^
Neoadjuvant ADT yes/no	20/50	21/50	1.000^†^
EBRT yes/no	17/53	17/54	1.000^†^
Pre use of alpha-1 antagonist yes/no	12/58	10/61	0.649^†^

Neo ADT: neoadjuvant androgen deprivation theraphy.

*Mann–Whitney U test.

EBRT: external beam radiation therapy.

^†^chi-square test.

Post-dosimetric parameters are shown in Table 2. There was not a significant difference (except prostate volume at post-dosimetry) between the naftopidil and silodosin groups

**Table 2:** **Post-dosimetric parameters**

	**Naftopidil (n=70)**	**Silodosin (n=71)**	**p-value**
	**Median (range)**	**Median (range)**	**(Mann–Whitney U test)**
PV (mL) at postdosimetry	26.8 (13.8-47.0)	29.1 (12.4-54.5)	0.031
%D90 (%)	114.1 (91.8-144.5)	115.5 (90.1-138.8)	0.410
D90 (Gy)	169.0 (110.6-213.5)	169.8 (115.9-218.5)	0.638
V100 (%)	95.8 (80.2-99.5)	96.0 (83.1-99.6)	0.378
V150 (%)	60.9 (33.6-87.9)	60.6 (32.5-84.4)	0.800
UD30 (Gy)	204.0 (123.7-267.4)	198.6 (133.0-276.6)	0.939
%UD30 (%)	134.8 (96.1-172.9)	137.9 (106.0-172.9)	0.833
UD90 (Gy)	141.2 (92.7-176.8)	139.1 (86.6-193.7)	0.673
%UD90 (%)	95.8 (63.4-149.2)	94.2 (67.2-121.0)	0.803
R100 (mL)	0.01 (0.00-1.00)	0.03 (0.00-1.00)	0.135
BED (Gy2)	197.7 (154.3-253.2)	202.5 (151.4-246.2)	0.635

PV: prostate volume at post-dosimetry, %D90: minimal percentage of the dose received by 90% of the prostate gland, D90: minimal does (Gy) received by 90% of the prostate gland, V100/V150: percentage of the prostate volume receiving 100% and 150% of the prescribed minimal peripheral dose, %UD30/UD30: minimal percentage of the dose and minimal dose (Gy) received by 30% of the urethra), %UD90/UD90: minimal percentage of the dose and minimal dose (Gy) received by 90% of the urethra, R100: rectal volume (mL) receiving 100% of the prescribed dose, and BED: biologically effective dose.

### IPSS and OABSS

The mean change in IPSS at 3 months after seed implantation in the two groups was ⊿10.6 in the naftopidil group and ⊿10.4 in the silodosin group. There was not a significant difference in the IPSS change between the two groups (p=0.728). The recovery rate of IPSS was 39% in the naftopidil group and 36% in the silodosin group 12 months after seed implantation. There was not a significant difference between the two groups (p=0.569).

The time-dependent change in IPSS showed a similar pattern in both groups (Table 3). A statistically significant increase in IPSS appeared at 1 month after seed implantation and lasted thereafter. The peak IPSS deterioration was observed at 3 months after seed implantation in both groups. Though IPSS moved to improvement at 6 and 12 months later, the mean IPSS was significantly higher than the baseline value after 12 months in both groups. The change pattern in OABSS with time was also similar to that of IPSS in both groups. A significant increase in OABSS appeared 1 month after seed implantation and lasted for 12 months in both groups (Table 3).

**Table 3:** **The serial change in mean value (SD) of IPSS and OABSS**

**Variable**	**Baseline**	**1 month**	**3 months**	**6 months**	**12 months**
***IPSS (total)***					
Naftopidil (n=70)	6.8 (4.3)	15.5** (8.0)	17.4** (8.5)	13.5** (7.9)	9.8** (7.1)
Silodosin (n=71)	7.9 (5.5)	15.5** (9.5)	18.3** (9.1)	14.4** (7.9)	9.8** (7.2)
Total (n=141)	7.4 (5.0)	15.5** (8.7)	17.8** (8.8)	13.9** (7.9)	9.8** (7.1)
***OABSS (total)***					
Naftopidil (n=70)	3.5 (2.2)	6.1** (3.4)	6.8** (3.8)	5.8** (3.2)	4.5** (3.0)
Silodosin (n=71)	3.4 (2.6)	6.2** (3.7)	6.4** (3.5)	5.2** (3.2)	4.0** (3.0)
Total (n=141)	3.4 (2.4)	6.2** (3.6)	6.6** (3.6)	5.5** (3.2)	4.3** (3.0)

Baseline vs. *p < 0.05, **p < 0.01.

### Uroflowmetric parameters

Voided volume remained at significantly decreased levels for 12 months after seed implantation in both groups. There was not a significant difference in voided volume between the 2 groups after seed implantation, except at baseline (260 mL in the naftopidil group vs. 209 mL in the silodosin group). Qmax was also significantly decreased throughout 12 months, showing no marked difference between the two groups. On the other hand, PVR increased significantly up to 6 months in both groups. The PVR at 12 months after seed implantation in the silodosin group was significantly greater than that in the naftopidil group (Table 4).

**Table 4:** **The serial change in mean value (SD) of uroflowmetric parameters**

**Variable**	**Baseline**	**1 month**	**3 months**	**6 months**	**12 months**
*Uroflowmetric parameters*					
**Vioded Vol.(mL)**					
Naftopidil (n=70)	260 (129)	153** (95)	141** (72)	155** (72)	185** (128)
Silodosin (n=71)	209 (111)	151** (89)	128** (81)	144** (76)	172** (100)
Total (n=141)	236 (123)	152** (92)	135** (77)	150** (74)	178** (115)
**Qmax (mL/s)**					
Naftopidil (n=70)	13.2 (6.0)	9.7** (4.4)	8.8** (3.8)	8.9** (3.3)	11.4** (5.4)
Silodosin (n=71)	12.1 (5.2)	9.4** (4.5)	8.7** (4.4)	9.5** (4.6)	11.0** (5.4)
Total (n=141)	12.6 (5.7)	9.6** (4.4)	8.7** (4.1)	9.2** (4.0)	11.2** (5.4)
**PVR (mL)**					
Naftopidil (n=70)	19 (38)	36** (45)	32** (39)	34** (38)	25 (36)
Silodosin (n=71)	23 (27)	38** (36)	52** (71)	37** (34)	35* (45)
Total (n=141)	21 (34)	37** (41)	42** (58)	35** (36)	30* (41)

*p < 0.05, **p < 0.01.

### Frequency volume chart

The total volume significantly decreased at 3 months after seed implantation and this was maintained up to 12 months in both groups. A significant difference in total urine volume was seen at 3 months between the 2 groups. The 24-hour urinary frequency showed a significant increase during 12 months after seed implantation. There was not a difference in the urinary frequency between the two groups. The mean voided volume significantly decreased during 12 months after seed implantation without any statistical differences between the two groups (Table 5).

**Table 5:** **The serial change in mean value (SD) of frequency volume chart**

**Variable**	**Baseline**	**1 month**	**3 months**	**6 months**	**12 months**
**Total volume (mL/day)**					
Naftopidil (n=70)	1915 (756)	1871 (745)	1782** (533)	1718** (652)	1687** (562)
Silodosin (n=71)	1777 (573)	1862 (610)	1569** (583)	1633** (634)	1652** (564)
Total (n=141)	1846 (672)	1866 (680)	1675** (576)	1676** (642)	1670** (561)
**24-hour urinary frequency**					
Naftopidil (n=70)	9.2 (2.4)	12.0** (3.9)	12.6** (3.9)	11.4** (3.7)	10.0** (2.5)
Silodosin (n=71)	9.2 (2.4)	12.3** (3.5)	11.8** (3.1)	11.2** (3.6)	10.3** (3.2)
Total (n=141)	9.2 (2.4)	12.2** (3.7)	12.2** (3.5)	11.3** (3.6)	10.1** (2.9)
**Mean voided vol (mL)**					
Naftopidil (n=70)	215 (84)	165** (66)	151** (60)	157** (58)	177** (71)
Silodosin (n=71)	199 (66)	159** (56)	140** (57)	153** (55)	168** (64)
Total (n=141)	207 (76)	162** (61)	145** (58)	155** (57)	173** (67)

*p < 0.05, **p < 0.01.

### Predictive variables of IPSS recovery at 12 months after seed implantation

We conducted univariate and multivariate analyses of the prediction of IPSS recovery at 12 months after seed implantation for the clinical parameters (age, neoadjuvant ADT, combination of EBRT, adjuvant ADT, prostate volume at post-dosimetry, IPSS at baseline, alpha-1 adrenoceptor antagonist) and postoperative dosimetric parameters. The multivariate analysis demonstrated that UD30 was an independent parameter predicting IPSS recovery at 12 months after seed implantation (Table 6). Using the 200 Gy cut-off point of UD 30, patients with UD 30 < 200Gy showed a 3 times higher rate of IPSS recovery at 12 months after seed implantation (p=0.002, odds ratio=3.094, 95% C.I.: 1.503-6.367).

**Table 6:** **The univariate and multivariate analyses predicting IPSS recovery at 12 months after seed implantation**

**Univariate**			**Multiivariate**		
	***p-*** **value**	**Odds ratio**	***p-*** **value**	**Odds ratio**	**95% C.I.**
Neo-ADT	0.081	0.510	n.s.		
EBRT	0.036	2.367	n.s.		
PV at post-dosimetry (mL)	0.066	0.957	n.s.		
D90(Gy)	0.018	0.984	n.s.		
UD30(Gy)	0.008	0.986	0.006	0.986	0.957-0.996

Neo ADT: neoadjuvant androgen deprivation therapy, EBRT: external beam radiation therapy, PV: prostate volume, D90: minimal dose (Gy) received by 90% of the prostate gland, UD30: minimal dose (Gy) received by 30% of the urethra.

## Discussion

LDR-brachytherapy has now become one of the definitive treatment modalities for prostate cancer alongside radical prostatectomy and IMRT, not only for low-risk patients, but also for intermediate and high-risk patients [11]. The decision making about the primary therapy for localized and advanced prostate cancer depends on the patients’ quality-of -life, their preferences, and the status of urinary and bowel conditions before treatment. Most patients experienced urinary disorders, especially urinary urgency and frequency up to 6–12 months after seed implantation [6-8]. It is an essential and important issue to prevent urinary adverse events in patients who undergo LDR-brachytherapy. Long ago, many investigators reported the efficacy of alpha-1 adrenoceptor antagonists to relieve postoperative urinary adverse events in patients who had undergone LDR-brachytherapy.

There were three phase 3 studies of alpha-1 adrenoceptor antagonists to prevent urinary adverse events in patients who had undergone LDR-brachytherapy [12-14]. First, Elshaikh *et al.* reported a randomized placebo-controlled study on the prophylactic effect of tamsulosin [12]. The mean IPSS at 5 weeks after seed implantation was significantly lower in the tamsulosin group, but there was not a significant difference in the acute urinary retention rate. Tsumura *et al.* conducted a direct comparison of 3 different types of alpha-1 adrenoceptor antagonists (tamsulosin, silodosin, and naftopidil). They concluded the superiority of alpha-_1A_ antagonist to change the IPSS compared with the alpha-_1D_ antagonist [13]. Shimizu *et al.* reported a controlled randomized study that compared a super-selective alpha-_1A_ adrenoreceptor antagonist group with a control group. The change in IPSS at 6 months after seed implantation and the change in the QOL score at 3 months after seed implantation in the alpha-_1A_ antagonist group were significantly smaller than those in the control group. However, there was not a significant improvement in the bladder outlet obstruction index (BOOI) in the silodosin group based on a pressure flow study [14]. On the other hand, the present study did not show a significant difference in the IPSS change between the alpha-_1A_ antagonist group and the alpha-_1D_ antagonist group. Generally, a significant increase in IPSS after seed implantation was seen in each study. Although a statistical improvement in IPSS was seen, the substantial difference between these studies was clinically subtle. Alpha-1 adrenoceptor antagonists act effectively to relieve urinary adverse events after seed implantation. However, the usefulness of these alpha-1 adrenoceptor antagonists has not been clarified sufficiently.

A phase 3 study was conducted to investigate whether anti-inflammatory treatment after seed implantation was useful for to relieve the adverse symptoms of brachytherapy [15]. They evaluated the effect of a cyclooxygenase-2 (COX-2) inhibitor. A 4-week course of meloxicam, starting either on the day of implant or 1 week prior to implant was evaluated. There was not a significant difference between the 2 arms. The 1-month edema of the prostate, IPSS at 1 and 3 months, and acute urinary retention rate did not show any differences.

The present study is the first report of FVC data after seed implantation. There were no obvious differences in the total urination volume, frequency of urination, or mean voided volume between the silodosin group and the naftopidil group. Overall, the total urination volume significantly decreased from 3 months up to 12 months after seed implantation, and the frequency of urination significantly increased up to 12 months after seed implantation. On the other hand, the mean voided volume significantly decreased up to 12 months after seed implantation. These changes showed a similar result when compared with IPSS and OABSS change as subjective parameters and uroflowmetric variables (voided volume, Qmax and PVR) as objective parameters.

We conducted multivariate analysis to predict the IPSS recovery at 12 months after seed implantation. Finally, the urethral dose (UD30) remained as a predictive parameter, while the use of an alpha-1 adrenoceptor antagonist, the combination of EBRT, the use of neoadjuvant androgen deprivation therapy, prostate volume, and the dose to the prostate (D90) did not remain. A higher dose to the urethra resulted in lower recovery of the IPSS (Table 6). Furthermore, patients with UD 30 < 200Gy showed a 3 times higher recovery rate in the IPSS at 12 months after seed implantation (p=0.002, odds ratio=3.094, 95% C.I.: 1.503-6.367). These results indicated that the prevention of excessive urethral irradiation is an important task to relieve the urinary condition of patients who undergo LDE-brachytherapy.

## Conclusion

Our present study could not detect any difference in the IPSS change between 2 different types of alpha-1 adrenoceptor antagonists. We however revealed, for the first time as far as we know, an increase in the urination frequency, and a decrease in the total urination volume and mean voided volume in the frequency volume chart up to 12 months after seed implantation. A lower dose to the urethra was an independent predictor of a higher recovery rate in the IPSS 12 months after seed implantation.

## Abbreviations

LDR-brachytherapy: Low-dose-rate brachytherapy
IMRT: Intensity modulated radiation therapy
ADT: Androgen deprivation therapy
IPSS: International prostate symptom score
EBRT: External beam radiation therapy
OABSS: Overactive bladder symptom score
Qmax: Maximum flow rate
PVR: Post voided residual
FVC: Frequency volume chart
%D90/D90: Minimal percentage of the dose and minimal dose (Gy) received by 90% of the prostate gland
V100/150: Percentage of the prostate volume receiving 100% and 150% of the prescribed minimal peripheral dose
%UD30/UD30: Minimal percentage of the dose and minimal dose (Gy) received by 30% of the urethra
%UD90/UD90: Minimal percentage of the dose and minimal dose (Gy) received by 90% of the urethra
R100: Rectal volume (mL) receiving 100% of the prescribed dose
BED: Biological effective dose
